# From Monocytes to M1/M2 Macrophages: Phenotypical vs. Functional Differentiation

**DOI:** 10.3389/fimmu.2014.00514

**Published:** 2014-10-17

**Authors:** Paola Italiani, Diana Boraschi

**Affiliations:** ^1^Laboratory of Innate Immunity and Cytokines, Institute of Protein Biochemistry, National Research Council, Napoli, Italy

**Keywords:** monocytes, monocyte-derived macrophages, tissue-resident macrophages, functional phenotypes, inflammation

## Abstract

Studies on monocyte and macrophage biology and differentiation have revealed the pleiotropic activities of these cells. Macrophages are tissue sentinels that maintain tissue integrity by eliminating/repairing damaged cells and matrices. In this M2-like mode, they can also promote tumor growth. Conversely, M1-like macrophages are key effector cells for the elimination of pathogens, virally infected, and cancer cells. Macrophage differentiation from monocytes occurs in the tissue in concomitance with the acquisition of a functional phenotype that depends on microenvironmental signals, thereby accounting for the many and apparently opposed macrophage functions. Many questions arise. When monocytes differentiate into macrophages in a tissue (concomitantly adopting a specific functional program, M1 or M2), do they all die during the inflammatory reaction, or do some of them survive? Do those that survive become quiescent tissue macrophages, able to react as naïve cells to a new challenge? Or, do monocyte-derived tissue macrophages conserve a “memory” of their past inflammatory activation? This review will address some of these important questions under the general framework of the role of monocytes and macrophages in the initiation, development, resolution, and chronicization of inflammation.

## Introduction

In the healthy organism, the innate immune system provides the first line of defense against external or internal danger signals, by initiating a protective inflammatory response that develops during time through different phases, from initiation and full inflammation, to resolution and re-establishment of tissue integrity. The first phase of an inflammatory response is aimed at destroying pathogens, and is followed by a phase in which dead and dying cells, damaged extracellular matrix material, and cellular debris are removed, to end up with a recovery phase in which the tissue is repaired and restored to a healthy fully functional condition. In fact, if the defense against harmful threats is a priority for avoiding tissue damage, maintaining homeostasis (i.e., maintaining tissue morphology and tissue function) is the ultimate goal of a tissue in multicellular organisms (1). In this perspective, inflammation presumably evolved as an adaptive response to tissue malfunction or homeostatic imbalance (2). Thus, while the disease state is a displacement from homeostasis, inflammation is the tissue response for restoring homeostasis. However, since the inflammatory activities are potentially harmful to the host, these need to be tightly controlled to avoid excessive tissue damage (3).

The mononuclear phagocyte system (MPS) plays major roles in development, scavenging, inflammation, and anti-pathogen defenses, both by the direct elimination of foreign agents and in organizing each different phase of the inflammatory process (4). Under the term, MPS are grouped lineage-committed bone marrow precursors, circulating monocytes, resident macrophages, and dendritic cells (DC) (5). The development, homeostatic maintenance, proliferation, differentiation, and function of the MPS are regulated by the growth factors colony-stimulating factor (CSF)-1 and interleukin (IL)-34, the second ligand for the CSF-1R (6, 7).

The issue of heterogeneity in the MPS still leads to a confusion and debate about DC as truly distinct cells from macrophages, with separate lineage and functions (8). In fact, macrophages and myeloid DC possibly represent alternative differentiation options of bone marrow progenitors and blood monocytes (9), with overlapping functions and marker expression. Reviewing this issue is beyond the scope of this essay [we refer the reader to recent excellent reviews on the topic; (9–11)], and will only focus on monocytes and their relationship with macrophages.

The traditional view of the MPS suggests that recruited monocytes (that become macrophages in tissues) are key players during inflammation and pathogen challenge, whereas tissue-resident macrophages have important roles in development, tissue homeostasis, and the resolution of inflammation. A basic concept of the MPS is that blood monocytes are precursors that replace tissue macrophages within a single developmental lineage (4). This dogma needs now to be revised in the light of new evidence that macrophages are endowed with self-renewal capacity and can populate tissues before birth, deriving from early hematopoiesis in the yolk sac (12, 13). The discovery of new macrophage progenitors of embryonic origin forces us to reassess definitions, functions, and cell–cell relationships within the MPS. We can synthesize it in three key new questions:

1. Are monocytes more than circulating precursors and can they have effector functions?
2. Is there a functional difference between the monocyte-derived macrophages and the yolk sac-derived self-renewing resident macrophages?
3. What is the relationship between monocytes and macrophages and which are their mutual roles in different phases of inflammatory reactions?

Another new perspective arises from the description of macrophage polarization, i.e., the ability of macrophages to acquire different functional phenotypes, enabling them to steer adaptive immunity in different directions. This highlights the central role of macrophages in immune defense, overturning the long-held notion that macrophages need to be activated by T-cells (14).

This review will summarize what has been so far investigated and established on monocyte/macrophage biology, highlighting what remains outstanding, and which questions are still unanswered. We will consider key studies that have been carried out in mice, with reference to the human situation when data are available. We will review the various aspects (monocyte recruitment, monocyte functions, macrophage polarization) before (homeostatic conditions), during (inflammatory reaction), and after a damaging event (resolution/repair).

## Monocytes

### Monocyte development and heterogeneity

Monocytes are a group of cells circulating in the blood, bone marrow, and spleen, and constituting ~10% of the total leukocytes in human beings and only 2–4% in mice. They have typical morphological features, such as irregular cell shape, oval- or kidney-shaped nucleus, cytoplasmic vesicles, and high cytoplasm-to-nucleus ratio. Monocytes can remain in the circulation for up to 1–2 days, after which time, if they have not been recruited into a tissue for facing a danger, they die and are removed. Monocytes originate in the bone marrow from hematopoietic stem cells (HSCs) and develop through a series of sequential differentiation stages: the common myeloid progenitor (CMP) (15), the granulocyte-macrophage progenitor (GMP) (15), the common macrophage and DC precursor (MDP) (16), and finally the committed monocyte progenitor (cMoP), a recently identified bone marrow precursor that differs from MDP as it lacks CD135 expression (17). MDP gives rise also to common DC progenitors (CDP), whose differentiation potential is restricted to the DC lineage (18). Monocytes have been considered as the systemic reservoir of myeloid precursors for renewal of tissue macrophages and DC. However, many DC and macrophage subpopulations [for example, lymphoid organ DC, plasmacytoid DC, skin Langerhans cells (LC), and brain microglia] originate from the MDP independent of monocytes (11, 18), and in some cases, they can even develop directly from the bone marrow (19).

Homeostatic control of monocyte/macrophage development is mostly influenced by CSF-1 (also known as M-CSF), produced by stromal cells within the blood and in tissues (20). Mature mononuclear phagocytes in turn express CSF-1 receptors (CSF-1R) and remove circulating CSF-1, allowing a feedback loop responsible for monocyte proliferation decrease (21, 22). Recently, the cytokine IL-34 has been identified as able to bind and signal through the CSF-1R (6, 23). Unlike broadly expressed CSF-1, IL-34 expression is restricted to the epidermis and central nervous system (24), where it supports the steady-state proliferation of macrophages (LC and microglia, respectively). Granulocyte-macrophage colony-stimulating factor (GM-CSF) is another factor involved in the development of mononuclear phagocytes but only during the inflammatory state and not under homeostatic conditions (25, 26).

Abundant experimental evidence indicates that recruited monocytes are innate effectors of the inflammatory response to microbes, and they kill pathogens via phagocytosis, production of reactive oxygen species (ROS), nitric oxide (NO), myeloperoxidase, and inflammatory cytokines (27). In some circumstances, monocytes can trigger and polarize T-cell responses (27, 28) and may also contribute to angiogenesis and atherogenesis (29).

Human peripheral blood monocytes are not a homogeneous population. Monocyte heterogeneity was first reported with the identification of a minor population of CD16 (FcγRIII)-expressing cells within circulating human monocytes (30). In recent years, investigators have identified three functional subsets of human monocytes, the characterization of which is still in its infancy. Likewise, it is still unclear which are the specific roles that they exert in homeostasis and inflammation *in vivo*, in comparison with those of the previously described classically and alternatively activated macrophages (see below). The new nomenclature that groups monocytes into three subsets, based on the expression of the surface markers CD14 and CD16, has recently been approved by the Nomenclature Committee of the International Union of Immunologic Societies (31). Based on this nomenclature, the major population of human monocytes (90%) with high CD14 but no CD16 expression (CD14^++^CD16^−^ or CD14^+^CD16^−^) are termed classical monocytes, whereas the minor population of human monocytes (10%) is further subdivided into the intermediate subset, with low CD16 and high CD14 (CD14^++^CD16^+^ or CD14^+^CD16^+^), and the non-classical subset, with high CD16 but with relatively lower CD14 expression (CD14^+^CD16^++^ or CD14^dim^CD16^+^) (31). In this review, we refer only to the main difference, terming classical monocytes simply as CD14^+^, and non-classical as CD16^+^.

Over the recent years, an increasing amount of knowledge has been gained in the field of monocyte subpopulations. Many authors demonstrated that the three subsets express different transcriptomes (32–38), although discrepancies between studies were evident. These discrepancies may be due to differences in cell isolation methodology and in the purity of the cell populations isolated, and the microarray methodologies, which use different amounts of total RNA for the hybridization, different probes to identify the genes, and even distinct solid supports for the probes (39). However, there is stronger agreement for the proximity of relationship between the intermediate and non-classical monocyte subsets, while the classical subset is the most distant subset (36). The close relationship between intermediate and non-classical monocytes suggests a direct developmental relationship between them, although this has yet to be formally proven. Also, it needs to be clarified how the characteristics previously ascribed to CD16^+^ monocytes are distributed between intermediate and non-classical subsets (36). Recent data suggested a sequential developmental relationship between the two subsets based on the observation that, in time course studies in inflammatory diseases, an increase in the intermediate monocytes is followed by an increase of non-classical monocytes (40).

The physiological role of the monocyte subsets *in vivo* is not fully defined. They might have different roles during the homeostasis, immune defense/inflammation, and tissue repair, in terms of their capacity to become activated and secrete inflammatory cytokines in response to different stimuli, antigen processing and presentation, pro-angiogenic and patrolling behavior. The phenotypic and functional differences between the monocyte subsets were recently discussed in an exhaustive review (41). The authors of this review report a complete and referenced list of studies on bacterial and viral infections, autoimmune diseases, and inflammatory conditions, in which an expansion of CD16^+^ cells in respect to other subsets has been observed. In general terms, both human classical and intermediate monocytes have inflammatory properties reminiscent of the murine Ly6C^+^ monocytes (also termed “inflammatory” monocytes) (42), while non-classical monocytes display patrolling properties similar to those of murine Ly6C^−^ monocytes (also termed “alternative” or “patrolling” monocytes) (43). Both human and mouse inflammatory monocytes express high levels of the chemokine receptor CCR2 and low levels of the chemokine receptor CX3CR1, whereas patrolling monocytes show a reverse pattern. Accordingly, inflammatory monocytes respond to the chemokine CCL2 that mediates Ly6C^+^/CD14^+^ monocyte recruitment to inflammatory sites (44), while patrolling monocytes respond to CX3C-chemokine ligand 1 [CX3CL1, the human fractalkine and mouse neurotactin; (45)], a chemokine present both as soluble protein and as membrane-bound chemokine form that is expressed on endothelial cells and in tissues. Overall, it is clear that the subsets between human being and mouse are similar but not identical (42, 46). Table 1 summarizes the main features of monocytes in human beings and mice. Of note, there is a clear difference in the proportion of the two monocyte subsets, as Ly6C^−^ cells represent about half of the circulating monocytes in mice, whereas CD16^+^ monocytes account for less than 15% in human beings (30). However, Ziegler-Heitbrock hypothesized that the higher proportion of the Ly6C^−^ in mouse blood could be due to stressful blood drawing (cardiac puncture under terminal anesthesia) that mobilizes these monocytes from the marginal pool (46). This hypothesis still needs experimental proof.

**Table 1:** **Human and murine monocyte subsets**.

Species	Subset^a^	% In WB	% In blood monocytes	Half-life	Markers	Chemokine receptors	Other surface markers	Main functions
Human being	Classical	~10%	85%	1–2 days	CD14^++^CD16^−^	CCR2^+^CX3CR1^−^	CD62L^+^, CD64^−^, MHC class II^+^, CD163^+^	Phagocytosis, inflammatory effectors
	Intermediate		5%	–	CD14^++^CD16^+^	CCR2^−^CX3CR1^+^	CD62L^+^, CD64^−^, MHC class II^++^, CD163^+^	Inflammatory effectors
	Non-classical		10%	–	CD14^+^CD16^++^	CCR2^−^CX3CR1^+^	CD62L^−^, CD64^+^, MHC class II^++^, CD163^−^	Patrolling, antiviral role
Mouse	Ly6Cow^low^	4%	~60%	18–20 h	CD11b^+^CD115^+^ Ly6C^+^	CCR2^+^CX3CR1^−^	F4/80^+^, CD62L^−^, MHC class II^b^, CD43^+^	Phagocytosis, Inflammatory effectors
	Ly6Cigh^high^		~40%	5–7 days	CD11b^+^CD115^+^ Ly6C^−^	CCR2^−^CX3CR1^+^	F4/80^+^, CD62L^+^, MHC class II^b^, CD43^−^	Patrolling, tissue repair

*^a^Work by Sunderkötter et al. (47) characterized a population of Ly6C^med^ monocytes with intermediate features between Ly6C^+^ and Ly6C^−^. These are not included in the table, because this population remains poorly characterized in terms of both phenotype and function*.

*^b^Inducible*.

*WB, whole blood; Ly6C, lymphocyte antigen 6 complex; CCR2, chemokine (C-C motif) receptor 2; CX3CR1, CX3C-chemokine receptor 1*.

To date, a relevant question that is still open concerns the origin of the various monocyte subpopulations. It should be kept in mind that the majority of current knowledge derives from mouse studies. It is unknown if the monocyte subpopulations are end stages of different differentiation paths of a common precursor, or whether they represent subsequent maturation stages in a common path of differentiation, where the intermediate subset could be a phenotypical and/or developmental intermediate between the classical and non-classical subsets. The latter hypothesis seems to be the most reliable. While initial studies suggested that Ly6C^+^ cells were recruited under inflammatory conditions and did not serve as precursors to Ly6C^−^ cells [which in turn were originally considered the immediate precursors of resident macrophages; (43)], recent evidence suggests that, in steady state, Ly6C^+^ monocytes are precursors of Ly6C^−^ monocytes (48, 49), as shown in experiments in which grafted Ly6C^+^ monocytes spontaneously differentiated into Ly6C^−^ in the blood of recipient mice (48). This conversion can also occur in the bone marrow, where Ly6C^+^ monocytes apparently return in the absence of inflammation (47–49). More recently, it has been suggested that CSF-1R signaling was required for the maturation of monocytes from Ly6C^+^ to Ly6C^−^, as blockade of this receptor leads to decrease in the number of Ly6C^−^ cells, shortens their lifespan (48), and concomitantly increases the number of Ly6C^+^ monocytes (50).

It has also been observed that development of the Ly6C^−^ population depends on the transcription factor NR4A1 (Nurr77) (51). NR4A1 deletion alters the number of Ly6C^−^ monocytes in the bone marrow but not in blood or spleen (52), but does not alter the number of macrophages within tissues (53). This suggests that either Ly6C^−^ monocytes can develop from MDP within the bone marrow, or that Ly6C^−^ monocytes are a functional end stage. In this regard, given that Ly6C^−^ monocytes exhibit a long steady-state half-life of 5–7 days [which in the absence of their renewal from Ly6C^+^ monocytes can extend to 2 weeks; (48)] compared to ~8 h for Ly6C^+^cells, Ly6C^−^ monocytes might be considered as terminally differentiated blood-resident macrophages or “vasculature macrophages,” rather than *bona fide* monocytes (48). Indeed, the primary function of these cells seems to be that of patrolling the vascular endothelium and monitoring its integrity (45, 51). Conversely, and in parallel with human CD14^+^ cells, Ly6C^+^ monocytes because of their short half-life are unlikely to have other functions, and thus are more likely to be the direct precursors of the tissue macrophages/peripheral mononuclear phagocytes described in the original MPS model.

### Function of monocyte subsets during homeostasis (classical vs. antigen-presenting tissue monocytes vs. patrolling monocytes)

The original concept of MPS implicated that classical monocytes are recruited in the tissue to become tissue-resident macrophages in homeostatic conditions, and inflammatory activated macrophages during an infection (27, 54). We will examine more in detail the role of recruited cells during the inflammatory response later, while here we will focus on the recruitment of monocytes in homeostasis and their contribution to maintaining the pool of tissue macrophages. In order to avoid misunderstandings, it is important to agree on the definition of monocyte. In our view, *bona fide* monocytes are restricted to the blood compartment, and to the bone marrow and spleen (55), where they wait to be released in the blood. For obvious reasons, in both these compartments, monocytes should not initiate any inflammatory reaction, but they must be ready to be recruited into the blood first and subsequently to all organs and tissues. A phenomenon was recently reported, termed “anticipatory inflammation,” whereby Ly6C^+^ classical monocytes are released from the bone marrow in diurnal rhythmic waves under the control of circadian gene *Bmal1* (or *Arntl*) (56) to provide an adequate innate response to environmental challenges that are expected to occur with a evolutionarily predicted frequency. Despite new evidence supports the view that Ly6C^+^ classical monocytes are not precursors of resident macrophages in all tissues and during certain types of inflammation (see below), it is clear that circulating monocytes contribute to the repopulation of tissue-resident macrophages under homeostatic conditions in tissues like the *lamina propria* of the small intestine and healthy skin. Studies based on functional and lineage tracing and adoptive transfer have revealed that Ly6C^+^ monocytes are precursors of intestinal macrophages that have a short half-life of only 3 weeks (57–59). Conversely, in the dermis are present both resident dermal macrophages and monocyte-derived macrophages (60, 61). A recent work suggests that the number of macrophages is partially replenished by monocytes also in the heart (62) and in the lung (63). It is unknown why some tissue macrophages are constantly maintained by circulating monocytes, whereas other populations are independent on circulating monocytes (see below). The notion that monocyte-derived macrophages derive from Ly6C^+^ cells suggests that the repopulation/maintenance of resident macrophages in steady-state conditions follows the same mechanism as that occurring during inflammation.

The function of Ly6C^+^ monocytes in circulation remains poorly defined. In the attempt to identify an effective role of monocytes in the blood in homeostatic conditions (besides being precursor cells), a recent work has suggested a distinct surveillance phenotype for Ly6C^+^ monocytes (64). These monocytes can enter non-lymphoid organs without obligatory differentiation into macrophages or DC. The authors propose that these monocytes can upregulate MHC class II expression and subsequently recirculate to lymph nodes, where they are able to present antigens to T-cells. Considering that these cells retain a monocyte-like gene expression profile, the authors term them “tissue monocytes” (64). This study contributes to revising the role of circulating monocytes, suggesting that they are not only precursors of macrophages but also effector cells.

Regarding the role of the Ly6C^−^ subset in the blood in steady-state conditions, intravital microscopy studies have established that these cells display a “patrolling” phenotype, being able to crawl on the luminal surface of the vascular endothelium (45, 51). This patrolling behavior, along with the ability to phagocytose endothelial-associated particles, suggests that a primary role of these monocytes is sensing and scanning the endothelial surface for damage and/or the presence of pathogens (51). The patrolling monocytes mainly respond via Toll-like receptor 7 (TLR7) to local danger signals (while they are poorly responsive to bacterial products such as LPS) by producing inflammatory mediators (51). They are able to induce the intravascular recruitment of neutrophils, which trigger endothelial necrosis, and subsequently they clear the resulting debris (51). A similar patrolling feature and TLR7/TLR8-dependent reactivity were also detected in human CD14^+^CD16^++^ monocytes (35).

Consistent with their functional role of surveillance of the endothelium integrity and with the fact that they are terminally differentiated cells, we agree with the view that Ly6C^−^/CD16^+^ can be considered as the tissue-resident macrophages of the blood. Regarding their ability to produce inflammatory factors, we speculate that the patrolling monocytes have a higher activation threshold than Ly6C^+^ monocytes; therefore, they should be able to produce an amount of inflammatory cytokines and chemokines sufficient for coordinating the repair of a damaged endothelium, but not enough to initiate a strong inflammatory reaction.

A summary of the roles of monocyte subsets in steady state vs. inflammatory conditions is reported in Table 2.

**Table 2:** **Functions of monocyte subsets in steady state and inflammatory conditions**.

Subset	Function	
	Steady-state conditions	Inflammation
Ly6C**^+^**	Replenishment of monocyte-derived macrophages in the tissue (gut, skin, heart, and lung)	Differentiation in M1-like functional phenotype and initiation of the inflammatory response
	Differentiation in Ly6C^−^cells in the blood and in the bone marrow	Antigen uptake in the tissue, recirculation to lymph nodes, antigen presentation in lymph nodes (“tissue monocytes”)
Ly6C^−^	Patrolling and surveillance of the luminal surface of the endothelium	Promotion of healing in ischemic myocardium, and tissue repair during infection with *Listeria monocytogenes*
	Sensing viral nucleic acids	

## Macrophages

### Tissue-resident macrophage development in steady state: embryonic origin vs. monocyte derivation

Resident macrophages are heterogeneous and versatile cells found in virtually all tissues of adult mammals, where they can represent up to 10–15% of the total cell number in quiescent conditions. This number can increase further in response to inflammatory stimuli. The specialization of macrophages in particular microenvironments explains their heterogeneity. Macrophages take different names according to their tissue location, such as osteoclasts (bone) (see 1), alveolar macrophages (lung), microglial cells (CNS), histiocytes (connective tissue), Kupffer cells (liver), and LC (skin). These populations have such highly different transcriptional profiles that they could be considered as many different and unique classes of macrophages (74). On the other hand, the functions of macrophages are the same in all tissues. They are key players in tissue development (by shaping the tissue architecture), in immune response to pathogens (by generating and resolving the inflammatory reaction), in surveillance and monitoring of tissue changes (by acting as sentinel and effector cells), and especially in maintenance of tissue homeostasis (by clearing apoptotic or senescent cells, and by remodeling and repairing tissues).

Box 1 **A hint on osteoclasts.**

Osteoclasts are multinuclear giant cells with a hematopoietic origin, commonly known as bone macrophages. They function in bone resorption and are involved in a normal skeletal development, growth, and modeling, for the maintenance of its integrity throughout life, and for remodeling through calcium metabolism (65). Moreover, osteoclasts are able to interact with the hematopoietic system and the adaptive immune system (66). Excessive bone loss mediated by osteoclasts plays a major role in certain pathologic conditions, such as rheumatoid arthritis (RA) and osteoporosis (67, 68). On the other hand, insufficient bone resorption due to the lack of functional osteoclasts (as in CSF-1R knock-out mice) leads to excessive bone apposition and osteopetrosis (69).

Osteoclasts really seem a class of macrophages on their own. They are generated from mononuclear phagocyte lineage progenitors in the bone marrow, and their differentiation from an osteoclast precursor (PreOC) depends on CSF-1 and the engagement of receptor activator of nuclear factor-κB (RANK) and its ligand (RANKL), a specific osteoclast differentiation factor (70). Recently, it has been shown that also IL-34 is involved in the osteoclast development (71).

Osteoclasts can differentiate *in vitro* from a cell population named monocyte-derived multipotential cells (MOMCs), which seem to originate from circulating CD14^+^ monocytes (72). *In vitro* induction of MOMCs from circulating CD14^+^ monocytes apparently requires their binding to fibronectin, and exposure to soluble factor(s) derived from peripheral blood CD14^dim^ monocytes (72).

Thus, culture of unfractionated peripheral blood monocytes with M-CSF and RANKL is sufficient to induce their differentiation into osteoclasts, and it has been assumed that osteoclast precursors are monocytes, although this has not been shown *in vivo*.

The question arises as to why osteoclasts, unlike other macrophages, have their own lineage of commitment and differentiation. Possibly, the reason may lie in the fact that they are phylogenically closely linked to the presence of bone, a tissue that develops late as compared to other organs and tissues during embryonic/fetal development, as in fact vertebrates are the most recent phylogenic step in the evolution (73).

The view that tissue macrophages originate from circulating peripheral blood monocytes that migrate into tissues under a variety of stimuli, proposed and strongly supported by van Furth in the 1970s (4, 75, 76), needs to be reconsidered. In addition to a wealth of old data (77, 78), two new pieces of evidence have further weakened the view that monocytes are the precursors of tissue macrophages in steady-state conditions: (1) the finding of the macrophage origin from embryonic progenitors that seed developing tissues before birth and give rise to fetal tissue macrophages (79) and (2) the self-maintaining ability of tissue-resident macrophages through local proliferation in adulthood (13). The latter finding will be discussed hereafter.

Two main phases of embryonic hematopoiesis have been described in the mouse: primitive hematopoiesis and definitive hematopoiesis. The former takes place in the ectoderm of the yolk sac and gives rise to macrophages without going through a monocytic progenitor. The latter takes place in the fetal liver, which is initially seeded by hematopoietic progenitors from the yolk sac and subsequently by HSCs from endothelium of the aorta-gonads-mesonephros (80, 81). The fetal liver subsequently becomes the source of definitive hematopoiesis that generates circulating monocytes during embryogenesis. Spleen and bone marrow are also colonized via the circulatory system by hematopoietic progenitors that will ultimately differentiate there. After birth, upon bone formation, hematopoiesis passes from the fetal liver to the bone marrow. The definitive bone marrow hematopoiesis is the source of both Ly6C^+^ and Ly6C^−^ circulating monocytes, from which resident tissue macrophages were thought to derive (10).

**Figure 1:**
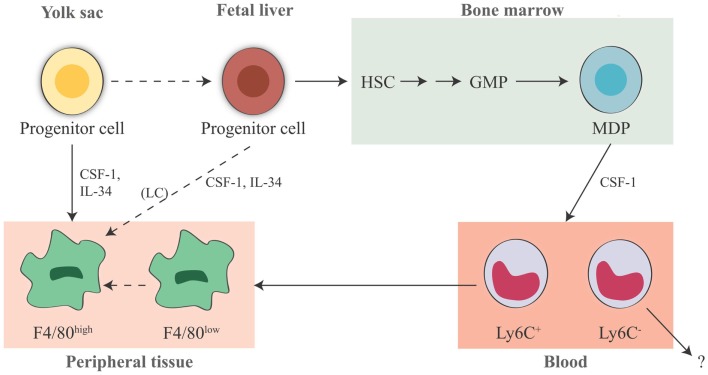
**Origin of tissue-resident macrophages in the mouse**. In adult tissues, macrophages derive from three sources. The first is the yolk sac in the embryo, where primitive hematopoiesis occurs giving rise to progenitors that seed tissues with F4/80^high^ macrophages. Later during fetal development, hematopoiesis shifts from the yolk sac to the fetal liver (that seems to contribute to the LC pool in the skin, possibly through a yolk sac-derived progenitor). It is unknown whether other resident macrophages in other tissues may also derive from fetal liver hematopoiesis. The third source is the bone marrow, where definitive hematopoiesis occurs in the fetus and in the adult, giving rise to monocytes and to monocyte-derived F4/80^low^ macrophages. Expression of murine F4/80 (the human EMR1) is an insufficient marker to discriminate between monocyte-derived macrophages and tissue-resident macrophages. It seems that Ly6C^+^ monocytes are the precursors of tissue macrophages, while the exact contribution of Ly6C^−^ monocytes remains unclear. HSC, hematopoietic stem cell; GMP, granulocyte-macrophage progenitor; MDP, macrophage–dendritic cell progenitor; LC, Langerhans cell; CSF-1, colony-stimulating factor 1; IL-34, interleukin 34.

The human embryonic hematopoietic system is organized roughly in the same way as in the mouse (82), and early studies propose that macrophages could arise in the embryo independent of bone marrow progenitors in human beings [for more extensive reading, see Ref. (83, 84)]. In summary, macrophages in fetal and adult tissues derive from at least three sources: yolk sac (giving rise to some tissue-resident yolk sac-derived macrophages), fetal liver (giving rise to fetal liver-derived macrophages), and bone marrow (giving rise to tissue-resident bone marrow-derived macrophages and inflammatory bone marrow-derived macrophages, see below) (Figure 1). The primitive yolk sac-derived macrophages have two distinct characteristics: (1) their pattern of differentiation does not go through a monocytic intermediate state but they directly become mature macrophages in fetal tissues (85) and (2) unlike macrophages derived from definitive c-Myb-dependent hematopoiesis, they are independent of the transcriptional factor c-Myb during development, while depending on the transcriptional factor PU.1 (12).

**Figure 2:**
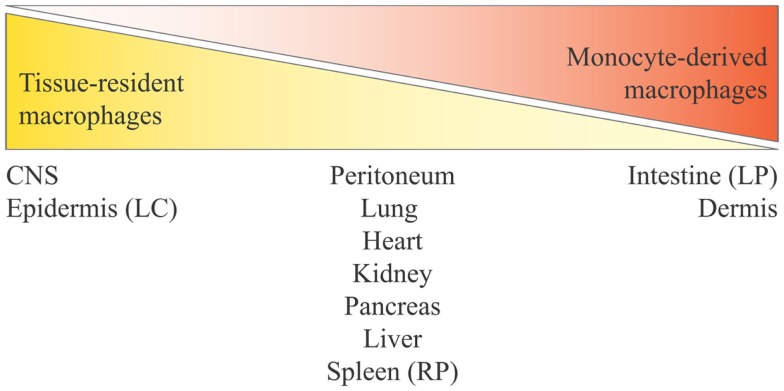
**Distribution of tissue-resident macrophages and monocyte-derived macrophages in tissues and organs**. Monocyte contribution to resident macrophages is highly tissue-dependent and varies from no contribution for brain microglia and epidermal LC to complete monocyte origin for intestinal *lamina propria* macrophages. The tissues listed in the middle are those at the center of ongoing controversy (see the main text), and for which a mixed contribution is probable. Here, we define yolk sac-derived macrophages as tissue-resident macrophages, and both fetal liver-derived macrophages and bone marrow-derived macrophages as monocyte-derived macrophages (considering that bone marrow hematopoiesis derives from fetal liver hematopoiesis). LC, Langerhans cells; LP, *lamina propria*; RP, red pulp.

Based on different experimental approaches, from lineage tracing (12, 48) to experiments carried out in parabiotic mice (64, 86), it is evident that monocytes do not contribute or contribute only minimally to the maintenance of peripheral tissue-resident macrophages in steady-state conditions in many adult tissues. Fate-mapping experiments have shown that the adult microglial cell population is exclusively derived from yolk sac progenitors (87, 88), whereas for LC in adult skin it was clearly demonstrated a mixed origin, from the yolk sac and from the fetal liver (12, 89). Moreover, using Myb-deficient mice that lack development of HSCs, followed by transplantation with genetically dissimilar bone marrow together with fate mapping, it has been observed that yolk sac macrophages can generate macrophages with a characteristically high expression of the F4/80 marker (F4/80 bright macrophages) in brain (microglia), skin (LC), liver (Kupffer cells), pancreas, and spleen (12). In kidney and lung, tissue-resident macrophages have a double origin, encompassing F4/80^high^ macrophages, derived from yolk sac, and F4/80^low^ macrophages, which have a hematopoietic origin and are continuously replaced by bone marrow-derived progenitors (12). Moreover, F4/80^high^ shares a common gene signature with yolk sac macrophages, unlike F4/80^low^ cells, as shown by global transcriptional analysis (12). Also, for splenic red pulp macrophages, alveolar, and peritoneal macrophages, an embryonic origin has been confirmed, rather than a monocyte origin (48). All these experiments show that early embryonic progenitor-derived macrophages can persist in tissues to adulthood. As mentioned previously, an exception is the gut, which contains a large population of resident macrophages that are all blood monocyte-derived cells, in steady-state conditions (57). How the mutual contribution of yolk sac-derived macrophages and fetal liver-derived monocytes is regulated in each tissue is unknown, and likewise it is not known how these two distinct populations of macrophages are functionally and ontogenically related. Regarding how much yolk sac progenitors contribute to originating adult tissue macrophages vs. fetal liver hematopoiesis, there are different opinions. One hypothesis is that fetal liver-derived monocytes proliferate and differentiate into adult tissue macrophages markedly diluting the population of yolk sac-derived macrophages (e.g., in lung and heart). This hypothesis stems from the observation that generation of yolk sac-derived macrophages does not go through a monocytic intermediate, therefore being in contrast with normal adult hematopoiesis, while a fetal liver origin for tissue macrophages would be reminiscent of the adult scenario in inflammation (90). Conversely, others believe that all tissue macrophages derive from yolk sac during the embryonic development, and circulating monocytes do not seed the majority of the adult tissues in mice (except kidney and lung) (12, 91) (Figure 2). This concept is strengthened by findings in human beings, where a complete loss of CD16^+^ monocytes seems to be of little consequence (92), and many tissue macrophage populations appear to be intact in patients with monocytopenia caused by immune deficiency syndromes (93, 94). In conclusion, to which extent different populations of yolk sac-derived macrophages may be later replaced by fetal liver-derived macrophages or monocytes, and how yolk sac-derived tissue-resident macrophages can proliferate locally through life to maintain their own pool independently of adult monocyte input, these issues remain a matter of debate (90, 91, 95). Thus, three main issues arise from all these findings:

1. The origin of adult macrophages in steady-state conditions can vary considerably between tissues.
2. The exact role of the patrolling Ly6C^−^ monocytes remains unclear, while Ly6C^+^ monocytes are recruited predominantly to sites of infection or injury, or to the organs and tissues that have continuous cyclic recruitment of macrophages (such as the uterus), or that are exposed to microbiota (such as the gut and the skin).
3. Due to some limits and weaknesses of the published studies (whole blood irradiation or other myelo-ablative treatments, parabiotic mice, engrafted bone marrow or monocytes, adoptive transfer of radiolabeled cells, Cre-*loxP*-based fate mapping, CCR2 or CSF-1 inactivation, etc.) (9, 90), none of such studies provides conclusive evidence against a role for monocytes in tissue macrophage homeostasis. Thus, more efficient and specific fate-mapping models of yolk sac-derived macrophages and fetal liver-derived monocytes are needed, along with further investigation, to determine which tissue macrophage populations are constantly replenished by circulating monocytes and which are not.

### Self-renewal/proliferation capacity of tissue-resident macrophages in homeostasis

Given that it is currently not possible to discriminate the two populations of tissue macrophages (yolk sac-derived and monocyte-derived) during homeostasis, we will report their ability to proliferate without considering them as distinct subpopulations. In any case, we will bear in mind the notion that the tissue macrophages can maintain their number in the absence of monocyte precursors both in steady-state conditions (12, 48, 64, 86) as well as in genetically or experimentally monocytopenic situations (94, 95).

It is important to clarify the difference between self-renewal and proliferative capacity. As stated by Sieweke and Allen (13), in immunology, self-renewal is understood as a replacement of a certain cell population, while in stem cell research as the capacity to generate with a cell division a daughter cell showing the same identity as the parental cell. Local proliferation of tissue macrophages can be considered as self-renewal in both senses [see Ref. (12, 13)], since macrophages can proliferate without change of their differentiated phenotype (96). Having said that, recent evidence demonstrated that macrophages within the adult tissues self-renew via proliferation in homeostatic conditions rather than through an influx of progenitors. This has been shown for LC, which are able to proliferate (97) both in human beings (98) and in mice (99), for brain microglia (19), resident peritoneal macrophages (100), and alveolar macrophages (101). The self-renewal process is regulated by growth factors and cytokines such as CSF-1 and GM-CSF (2).

Box 2 **Factors driving monocyte/macrophage self-renewal, proliferation, and functional differentiation.**

Macrophage colony-stimulating factor (M-CSF, also known as CSF-1) and granulocyte-macrophage colony-stimulating factor (GM-CSF) drive the monocyte/macrophage development, differentiation, and proliferation along with cytokines such as IL-4 (102) and the recently discovered IL-34 (103). Macrophages and circulating monocytes express the CSF-1 receptor (CSF-1R) (42, 43), and mouse deficient in CSF-1R (osteopetropic mice, *op/op*) have a decreased number of monocytes in the bone marrow and in circulation, in addition to a decrease in osteoclasts (69, 104). Experimentally blocking CSF-1R with antibodies leads to a reduction in Ly6C^−^ monocytes (69) and to an associated increase in Ly6C^+^ monocytes, suggesting the involvement of CSF-1 in the maturation of monocytes from Ly6C^+^ to Ly6C^−^ (48, 104). CSF-1 is constitutively produced by mesenchymal cells (105) and is detectable in circulation in resting conditions (20). Under homeostatic conditions (106), CSF-1 promotes monocyte development and macrophage proliferation (107), which is controlled in a negative feedback loop. In fact, mature mononuclear phagocytes express high level of CSF-1R and are responsible for the clearance of CSF-1. The decreased CSF-1 levels lead to a decrease in mononuclear cell proliferation, thereby maintaining the cell number to normal levels both systemically and locally [(21, 22); a model of CSF-1-dependent local homeostasis of macrophage density has been described by Jenkins and Hume (9)]. Thus, elevated production of CSF-1 can drive both an increased proliferation of resident macrophages and an increased recruitment of monocytes (103, 108) via macrophage production of CCL2 (109). CSF-1 deficiency in mice affects distinct tissues by different degrees, ranging from marked cell loss in the gut, kidney, peritoneal cavity, and in circulation, as compared to liver (86). CSF-1 is also involved in the proliferation of splenic red pulp macrophages and bone marrow macrophages (110). GM-CSF is also critical for macrophage homeostasis and proliferation, especially in the lung (111) and in the peritoneal cavity *in vivo* (112), but it is less important in hematopoiesis, and, therefore, for monocyte development (113). GM-CSF can support monocyte expansion and differentiation *in vitro* (25, 114), and it seems to be mainly involved in induction of hematopoiesis during inflammation rather than in homeostasis (115, 116). CSF-1 and GM-CSF are also involved in monocyte/macrophage functional differentiation programs: CSF-1 stimulation leads to a homeostatic or anti-inflammatory M2-like phenotype (25, 117, 118), whereas GM-CSF leads to an M1-like inflammatory phenotype (25, 117–119). Thus, CSF-1 stimulation represents a default homeostatic/M2 pathway of monocyte development (119). In summary, CSF-1 is mainly involved in self-renewal of tissue macrophages, consistent with its role in M2 polarization, while GM-CSF is involved in proliferation of monocyte-derived inflammatory macrophages, consistent with its role in M1 polarization.

Resident macrophages can proliferate at low levels in steady-state conditions, but proliferation rates strongly increase after macrophage depletion (86) or under inflammatory challenge (13). Regarding their proliferative ability, Ginhoux and Jung (90) raise the interesting question as to “whether all macrophages within a tissue possess equal self-renewal potential, or whether there are macrophage subpopulations that differ in their capacity of survival and proliferation, which would imply the existence of macrophages subpopulations with stem cell-like features.” The question arises from observations that physiological or experimental depletion of LC leads to a clonal expansion of LC by adjacent proliferative cell clusters with stem cell-like features (120), and that among lung macrophages, different cells can proliferate to maintain the population (87). To solve this issue, there is evidence that a macrophage that had previously divided has the same probability of entering the cell cycle as a cell that had not, suggesting the same proliferative ability for all macrophages (87). This is consistent with the observation that macrophages genetically modified to have an indefinite self-renewal potential can be efficiently cloned (96).

During inflammation, things are quite different, especially because the tissue is enriched with monocyte-derived macrophages. We will discuss later the replenishment of tissue macrophages by monocyte-derived macrophages and their ability to proliferate.

### Tissue macrophage functions

Table 3 summarizes the functions of resident macrophages in the main body tissues. These functions, mirroring different phenotypes (74, 143), are specific because depending on different tissue microenvironments. Different tissues define different phenotypes of both resident macrophages and monocyte-derived macrophages recruited from the reservoirs of blood, spleen, and bone marrow (10), phenotypes that are necessary for the tissue-specific needs of defending, maintaining, and regaining homeostasis (144). These homeostatic functions may be altered by chronic insults, which may lead to an anomalous prolongation/amplification of the macrophage attempt to regain homeostasis and to a consequent causal association between macrophages and diseases (Table 3). In pathological conditions, the distinction between tissue-resident macrophages and recruited inflammatory macrophages has not yet been possible. For an in-depth analysis of these issues, the reader can refer to recent exhaustive reviews (99, 144, 145). Apart from tissue-specific functions, tissue macrophages share a series of common functions encompassing clearance of cell debris, immune surveillance, wound healing, defense against pathogens, and the initiation and resolution of inflammation. In this review, we will only focus on the role of macrophages in inflammatory responses, considering their capacity to polarize into different functional phenotypes in response to the tissue microenvironmental changes that occur during the different phases of an inflammatory response. This polarization process is based on the M1–M2 paradigm (see below).

**Table 3:** **Macrophage functions and the pathological consequences of their anomalous activation in the main tissues**.

Macrophages (MΦ)	Tissue	Functions	Pathology
Microglia	Brain	Brian development (121), immune surveillance, synaptic remodeling (122)	Neurodegeneration (123)
Osteoclasts	Bone	Bone modeling and remodeling, bone resorption (124), support to hematopoiesis (125)	Osteoporosis, osteopetrosis, arthritis (126)
Heart MΦ	Heart and vasculature	Surveillance	Atherosclerosis (127)
Kupffer cells	Liver	Toxin removal, lipid metabolism, iron recycling, erythrocyte clearance, clearance of microbes, and cell debris from blood (128, 129)	Fibrosis (130), impaired erythrocyte clearance (131)
Alveolar MΦ	Lung	Surfactant clearance, surveillance for inhaled pathogens (132)	Alveolar proteinosis (133)
Adipose tissue-associated MΦ	Adipose tissue	Metabolism, adipogenesis, adaptive thermogenesis (134)	Obesity, diabetes, insulin resistance, loss of adaptive thermogenesis (131)
Bone marrow MΦ	Bone marrow	Reservoir of monocytes, waste disposal (131)	Disruption of hematopoiesis (131)
Intestinal MΦ	Gut	Tolerance to microbiota, defense against pathogens, intestinal homeostasis (135)	Inflammatory bowel disease (136)
Langerhans cells	Skin	Immune surveillance (137)	Insufficient healing, fibrosis (138)
Marginal zone MΦ, red pulp MΦ	Spleen	Erythrocyte clearance, iron processing, capture of microbes from blood (139)	Impaired iron recycling and erythrocyte clearance (140)
Inflammatory MΦ^a^	All tissues	Defense against pathogens, protection against dangerous stimuli (141)	Chronic inflammation, tissue damage, autoimmunity (91)
Healing MΦ^b^	All tissues	Branched morphology, angiogenesis (142)	Cancer, fibrosis, epithelial hyperplasia (91)

*^a^Also known as inflammatory macrophages or M1 macrophages*.

*^b^Also known as deactivated or M2 macrophages*.

## Plasticity of Monocytes/Macrophages during Inflammatory Reaction

### Monocyte recruitment during the inflammatory reaction: Inflammatory monocyte-derived macrophages vs. tissue-resident macrophages and their proliferative ability

During the first phases of an inflammatory reaction, there is in the tissue an increase of the effector cell number, necessary for increasing the immune defensive firepower. These cells are monocyte-derived macrophages. The concomitant drastic loss of resident macrophages, due to tissue adherence, emigration, or death, is a phenomenon termed “the macrophages disappearance reaction” (146), and it is especially evident for peritoneal and alveolar macrophages. To cope with the need of increasing the number of effector cells, two strategies come into play.

First is the recruitment of blood monocytes, driven by resident macrophages alongside with other tissue cells. Recruited blood monocytes are a source of inflammatory macrophages, which take the name of bone marrow-derived or monocyte-derived inflammatory macrophages. The other strategy is the increase of tissue-resident macrophage proliferation by enhancement of their self-renewal ability.

Central to the issue of monocyte recruitment is the difference in monocyte subset trafficking. Such differences have been observed to occur during acute and chronic inflammation in mice, and underline the fact that the monocyte subsets are under the control of distinct trafficking mechanisms, with the classical subset being recruited via CCR2 and the non-classical one utilizing a CXCR1-dependent pathway (see 3).

Box 3 **Chemokines and monocyte recruitment mechanisms.**

The two main chemokines and related receptors involved in the inflammation-dependent recruitment of the Ly6C^+^ and Ly6C^−^ monocyte subsets from the blood, bone marrow, and spleen, are CCL2/CCR2 and CX3CL1/CX3CR1, respectively (45, 46). Fibroblasts, epithelial, and endothelial cells produce CCL2 in response to inflammatory cytokines or microbial molecules, and generate a high level of this chemokine in the inflamed tissue (to allow egress of monocytes from the blood and entry in the tissues) and/or in blood (to allow entry of bone marrow monocytes) (44, 147). Recently, it has been proposed that both mesenchymal cells and progenitor cells closely apposed to bone marrow vessels can produce CCL2 in inflammatory situations, to allowing the egress of monocytes from the tissue and their subsequent entry into the blood (148). During a bacterial infection, Ly6C^+^ monocytes require CCR2 for being recruited from the bone marrow into the blood (149). In mice lacking CX3CR1, a reduction of patrolling by Ly6C^−^ was observed (45), and a reduction of their number in infracted heart (150), suggesting an impaired recruitment from the blood. Genetic destruction of CCR2 reduces the accumulation of both Ly6C^+^ and Ly6C^−^ monocytes in injured skeletal muscle, but it does not alter the recruitment of Ly6C^−^ monocytes in the heart after myocardial infarction (150). A reduction was also observed in skin wounds on the first day from injury, when Ly6C^+^ cells are those principally involved in the early repair phases, but not during the late stage of tissue repair, when Ly6C^−^ cells are dominant (151). These studies underline the importance of monocyte recruitment from blood to the tissue in the injured cardiac or skeletal muscle. Regarding the role of *in situ* differentiation, in addition to the data mentioned above (150), a reduction of Ly6C^−^ monocytes has been observed also in the blood of CCR2-deficient mice, despite the fact that they do not express this receptor (48). Macrophage accumulation in skin wounds is also reduced in mice lacking CX3CR1 (152). CX3CL1 and CX3CR1 provide a survival or anti-apoptotic signal to Ly6C^−^ cells (153). Two models have been proposed for the CCL2-dependent Ly6C^+^ cell recruitment from the bone marrow: CCL2 increases monocyte chemokinesis and contact with blood vessels; CCL2 associates with tissue glycosaminoglycans and forms a gradient driving monocytes to exit the bone marrow for entering into circulation (54). Intravenous administration of CCL2 leads to the mobilization of monocytes into the circulation, which is consistent with a role for peripheral CCL2 production responsible for replenishment of circulating monocytes from bone marrow (154).

Under steady-state conditions, the release of Ly6C^+^ and Ly6C^−^ monocytes from bone marrow depends on two genes, the circadian clock gene *Bmal1* for Ly6C^+^ cells (55), and the G-coupled receptor for sphingosine-1-phosphate S1PR5 for Ly6C^−^ monocytes (155). In mice with myeloid cell-restricted *Bmal*1-deficiency, the rhythmic release of CCL2 was ablated along with monocyte pools. Thus, myeloid cells produce low diurnal levels of CCL2 in a circadian fashion, and CCL2 in turn stimulates the release of CCR2-expressing monocytes from the bone marrow into the blood. On the other hand, in S1PR5-deficient mice, Ly6C^−^ monocytes are retained in the bone marrow and are not released in the blood and spleen.

In a model of *Listeria monocytogenes* infection, non-classical monocytes (Ly6C^−^) extravasate rapidly within 1 h, invade the surrounding tissues, and develop a very early inflammatory response by producing chemokines responsible for recruiting other effectors cells (granulocytes, NK cells, T-cells), and cytokines such as TNF-α (central to macrophage-mediated inflammation and innate responses) (45). This inflammatory response is transient, and 8 h after infection, the main producers of inflammatory cytokines in the tissue are the classical monocytes (Ly6C^+^). As previously mentioned (45), it has been observed that the two subsets of monocytes differentiate into two distinct cells types. Ly6C^−^ patrolling monocytes initiate a macrophage differentiation program that resembles that of M2 macrophages (see below), while Ly6C^+^ monocytes differentiate into DC-like cells that resemble Tip-DC (45). However, in other systems, this double recruitment of different monocyte subsets has not been observed. Only Ly6C^+^ monocytes were observed to migrate to the injured tissue in a model of skeletal muscle injury and be responsible for early inflammatory responses (156). Generally, classical monocytes infiltrate inflamed tissues more robustly than their non-classical counterparts, and their number is significantly increased in the circulation during systemic or chronic infection (27). After engulfing dying cells in the tissue, the recruited classical monocytes differentiate into cells that resemble Ly6C^−^ monocytes, and become involved in tissue repair mechanisms (156). Likewise, in a mouse model of sterile wound (subcutaneous polyvinyl alcohol sponge implantation), it has been recently demonstrated that Ly6C^+^ monocytes recruited from the circulation into the skin acquired an inflammatory function and, despite time of maturation was long, they matured into Ly6C^−^ macrophages with repair functions (157). Yet, another situation is that of myocardial infarction, during which both monocyte subsets appear to home to the same tissue at different stages of inflammation (150). Specifically, the Ly6C^+^ subset first infiltrates the infracted heart and exhibits inflammatory functions, while the Ly6C^−^ subset is recruited at a later stage and promotes tissue healing by expressing high amounts of vascular endothelial growth factor, exhibiting angiogenic capacity, and promoting deposition of collagen (150).

In atherosclerosis, as a model of chronic inflammation, both monocyte subsets are recruited at the same time to the activated endothelium/plaques, and healing seems to be correlated with a reduction in total monocyte recruitment (158). However, it was recently demonstrated that the maintenance and accumulation of monocyte-derived macrophages in atherosclerotic plaques mainly depend on local proliferation of bone marrow-derived macrophages rather than on the influx of circulating monocytes (127, 159). In an atopic dermatitis model and in experimental autoimmune encephalomyelitis, a massive proliferation of LC and microglia cells has been observed (160, 161), despite a significant monocyte influx (161). In the peritoneal cavity and in the lung, where the macrophage disappearance phenomenon occurs upon bacterial and virus insults, the few remaining macrophages are responsible for repopulating the tissue (86, 101, 106). Similarly, in the context of Th2-mediated immunity against nematode infection, IL-4 drives tissue-resident macrophage expansion in the pleural cavity in the absence of peripheral monocyte recruitment (102).

Proliferation of macrophages is observed in a variety of human diseases [see Ref. (91)], including tumor-associated macrophages in solid tumors (162), and adipose tissue-associated macrophages in obesity (163).

In this context, a question is still open. Having established that monocytes are recruited into tissues during an inflammatory event, to what extent are they capable to differentiate in tissue macrophages and to proliferate? As proposed by Jenkins and Hume, the negligible contribution of monocytes to the pool of resident macrophages could be due to the fact that monocyte recruitment is specifically aiming at providing a population of functionally differentiated cells needed for resolving an acute inflammatory event, rather than being triggered by the homeostatic need of maintaining the autonomous pool of resident macrophages (9). This view is supported by another interesting hypothesis, i.e., in inflammatory conditions, monocyte-derived macrophages are mostly end-type killer cells, as the non-specific toxic molecules they produce will also cause their own death (164).

The gastrointestinal tract provides evidence in favor of this hypothesis. In the gut, blood monocytes are constantly recruited to the tissue where they contribute to maintaining the resident macrophage population, but during an inflammatory event they re-program their differentiation plan toward adopting an inflammatory phenotype (57, 165).

Thus, we should consider that monocyte-derived macrophages adopt different and opposing phenotypes based on microenvironmental signals. Adoption of a phenotype or another depends on the time by which the sequential waves of recruited Ly6C^+^ monocytes reach the tissue during the course of the inflammatory reaction, since the incoming monocytes will find a different microenvironment in different phases of the reaction. In this context, it is conceivable that monocytes entering the tissue at later times could find conditions favorable to adopting an M2-like phenotype (see above), thereby becoming tissue macrophages over time.

Inflammatory monocyte-derived macrophages (12, 86) and tissue monocytes (64) can be phenotypically and functionally distinguished from resident macrophages in many tissues. In the central nervous system, inflammatory monocyte-derived macrophages do not contribute to the resident population (161). In contrast, fate-mapping experiments revealed that monocyte-derived macrophages recruited to the peritoneal cavity upon thioglycollate injection differentiate into resident macrophages and persist over time (48). The fraction of monocyte-derived macrophages that do not die upon inflammation and become tissue-resident macrophages share gene profiling with resident macrophages (45, 64, 165), but there is no information as to whether they are functionally different or not.

The accumulation of inflammatory monocytes in an inflamed tissue is due to their influx from blood rather than by their proliferative ability, and in fact inflammatory signals of microbial origin generally prevent their proliferation. An exception to this general paradigm comes from a recent study that has demonstrated that also inflammatory monocyte-derived macrophages can proliferate at certain stages during the resolution of zymosan-induced peritonitis (106).

**Figure 3:**
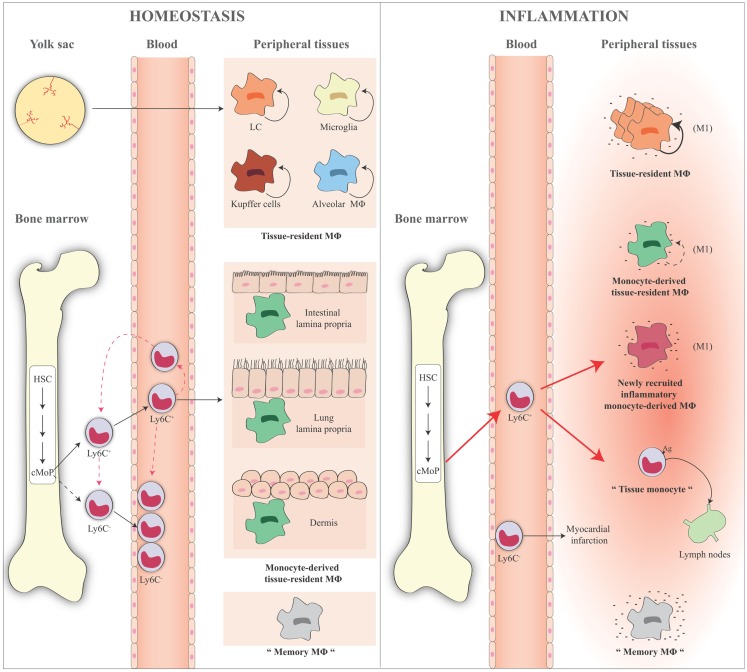
**Schematic representation of monocyte and macrophage populations in homeostasis and inflammation**. Under homeostatic conditions (left panel), Ly6C^+^ monocytes derive from the bone marrow and circulate via the blood into the tissue. A minor fraction of these cells lose Ly6C expression and become Ly6C^−^ monocytes in the blood or in the bone marrow where some of them might return in the absence of inflammation. Ly6C^+^ blood monocytes enter tissues and become either macrophages, for example, in the gut, lung, and dermis (monocyte-derived macrophages or monocyte-derived tissue-resident macrophages). Some tissue macrophages derive directly from yolk sac during the embryogenesis (e.g., LC, microglia, liver Kupffer cells, and alveolar macrophages), are long lived, and are mainly maintained by self-renewal (tissue-resident macrophages). Ly6C^−^ monocytes act as resident macrophages of the vasculature, patrolling, and monitoring the endothelial surface in the blood vessel lumen. In the figure, the presence of “trained” macrophages is also considered, which we define as “memory macrophages,” i.e., the tissue macrophages that retain the memory of a previous inflammation and are in a quiescent state in the tissue. During an inflammatory reaction (right panel), the number of blood Ly6C^+^ monocytes recruited to an inflamed tissue increases considerably. The large majority of these cells gives rise to the inflammatory monocyte-derived macrophages, while some of them do not differentiate into macrophages and remain monocyte-like cells, are able to take up antigens, and to migrate to the draining lymph nodes (tissue monocytes). These are the antigen-uptaking and -presenting cells of the tissue. During inflammation, all macrophages (tissue-resident macrophages, monocyte-derived tissue macrophages, inflammatory monocyte-derived macrophages) are activated and differentiate into M1-like inflammatory cells following interaction with pathogenic and damaged signals/insults in surrounding microenvironment. These cells produce a series of cytokines and other inflammatory factors. Tissue-resident macrophages increase their capacity of proliferation to compensate the loss of macrophages caused by the inflammatory reaction. Recent evidence demonstrates that also inflammatory monocyte-derived macrophages are able to proliferate in a late phase of the inflammatory reaction. Memory macrophages are important players in the inflammatory reaction, as they can react to inflammatory stimuli with a faster and stronger inflammatory cytokine production. The role of circulating Ly6C^−^ cells during an inflammatory reaction is not fully identified. They probably remain in the blood vessels as sentinels, and in some cases they could enter in the tissue, as it has been reported in the case of myocardial infarction, to take up a repair function. HSC, hematopoietic stem cell; cMoP, common monocyte progenitor; Ly6C, lymphocyte antigen 6 complex; LC, Langerhans cells; MΦ, macrophages.

All these findings are summarized in Figure 3.

Finally, two issues should be reminded:

1. The precise nature and extent of the contribution of monocyte-derived macrophages to tissue macrophages could depend on how, and to which extent, inflammation or its cause has affected the tissue-resident macrophages. In this view, as proposed by Ginhoux and Jung (90), tissue-resident macrophages are more involved in tissue macrophage repopulation after mild injury, while monocyte-derived macrophages are more involved in severe inflammatory injuries.
2. In the mouse it seems that non-classical monocytes contribute to the resident macrophage population. It is possible (although there is little evidence in this respect) that when non-classical monocytes are recruited in the inflamed tissue, they may differentiate into alternatively activated macrophages, while classical monocytes would give rise to classically activated macrophages. In this context, the developmental relationship between the different monocyte subsets and the different macrophage functional phenotypes has yet to be fully and formally proven. No evidence in this sense has been generated yet in human being (see below).

### Macrophage differentiation and functional phenotypes

Macrophage polarization occurs through different activation programs by which macrophages carry out their defensive functions. In this way, macrophages become able to respond with appropriate functions in distinct contexts, functional diversity becoming the key feature of these cells. Essentially, macrophages can modify their metabolic functions from a heal/growth promoting setting (M2 macrophages), to a killing/inhibitory capacity (M1 macrophages) (145, 166). The main difference between these cells is that in M2 macrophages the arginine metabolism is shifted to ornithine and polyamines, while in M1 cells it is shifted to NO and citrulline (166). M2-produced ornithine can promote cell proliferation and repair through polyamine and collagen synthesis, fibrosis and other tissue remodeling functions (167), while M1-produced NO is an important effector molecule with microbicidal activity and cell proliferation inhibitory capacity (168). Interestingly, polyamine production *per se* has been reported to be a driver of M2 polarization (169), and M2 is the normal “default” program adopted by resident macrophages (170). Moreover, M1 and M2 macrophages have distinct features in terms of chemokine production profiles (171), and iron and glucose metabolism (172, 173).

The description of macrophages polarization is leading immunologists to take a step back and revise their concept on how the immune system works (14, 145). The M1 and M2 definition was formulated by mirroring the Th1/Th2 polarization concept. However, this definition might be misleading in that it may suggest that Th1/Th2 cells do instruct M1/M2 polarization, whereas it is now obvious that the reverse is true, i.e., macrophages are initiating and directing T-cell polarization. Since it has been shown that distinct populations of macrophages promote and control CD4^+^ T-cell-dependent type 1 and type 2 immune inflammatory responses (i.e., those against viruses and bacteria, and against multicellular parasites, respectively), not surprisingly they have been termed M1 and M2 (166). Thus, considering that macrophages recognize pathogens directly (174, 175), while T-cell do not, and considering that T-cells proliferate through interaction with macrophages (176), it is logical to think that macrophages are the cells that initiate and direct T-cell response, and that the adaptive immune response needs the triggering and guidance of innate immunity (14). Notably, M1 and M2 macrophage activities do not need the presence of lymphocytes (145). The fact that T-cell-derived cytokines such as IFN-γ and IL-4 may amplify macrophage polarization (see below) should not deceive us into believing that macrophage polarization cannot occur without lymphocytes, as this is not the case. *In vitro*, macrophages are activated toward an M1 functional program by infectious microorganism-related molecules (e.g., the gram-negative product LPS) and by inflammation-related cytokines TNF-α or IFN-γ, alone or in combination. M1 macrophages are characterized *in vitro* by an IL-12^hi^IL-23^hi^IL-10^lo^ phenotype; are efficient producers of toxic effector molecules (ROS and NO) and inflammatory cytokines (IL-1β, TNF, IL-6); participate as inducers and effector cells in polarized Th1 responses; and mediate resistance against intracellular parasites and tumors (177). Conversely, M2-like polarization has been observed *in vitro* in response to the Th2-related cytokines IL-4 or IL-13 (178), to the concomitant triggering of Fcγ receptors and Toll-like receptors (TLR), to immune complexes, and to anti-inflammatory molecules such as IL-10, TGF-β, and glucocorticoids (115). The variety of functional programs adopted by macrophages in response to the stimuli listed above has been termed M2a (IL-4 and IL-13; alternative inflammation), M2b (immune complexes, FcγR/TLR triggering), and M2c (IL-10, TGF-β, glucocorticoids; deactivation) (178, 179). M2 cells are characterized *in vitro* by an IL-12^lo^IL-23^lo^IL-10^hi^TGF-β^hi^ phenotype and generally have high levels of scavenger, mannose, and galactose-type receptors. In general, these macrophages take part in polarized Th2 responses, allergy, parasites clearance, dampening of inflammation, tissue remodeling, angiogenesis, immunoregulation, and tumor promotion (180).

Macrophage taxonomy is an attempt to rationally categorize an extended variety of cell functions. Indeed, the M1/M2 paradigm is a limited attempt to define the complexity and plasticity of mononuclear phagocytes. *In vivo*, macrophages can adopt a variety of functional phenotypes depending on subtle and continuous changes in the tissue microenvironment. So, the M1/M2 polarization of macrophage functions may be taken as a simplified conceptual framework describing a continuum of diverse functional states, of which M1 and M2 activation states are not ontogenically defined subsets but represent the extremes (180–182). In this regard, Mosser and Edwards (181) have suggested a macrophage classification that takes into account the three functions of these cells in maintaining homeostasis: host defense, wound healing, and immune regulation. Classifying macrophages according to these functions provides three basic macrophage populations: classically activated macrophages, wound-healing macrophages, and regulatory macrophages (183, 184). The authors believe that this classification also helps to illustrate how macrophages can evolve to exhibit characteristics that are shared by more than one macrophage population (181).

Without going into details [for which we refer the reader to other reviews; (14, 185)], the M1/M2 classification in different subsets can create the illusory perception of a heterogeneity, which is not proven to exist *in vivo*. Thus, as already mentioned above, it is logical to hypothesize that the subsets are varying mixtures of M1- and M2-type macrophages, as observed in the lung and in the peritoneal cavity, where tissue-specific variations in the balance of M1- and M2-type responses have been revealed (74, 186). This situation has also been observed in pathological conditions, where macrophages can develop mixed M1 and M2 phenotypes (187, 188). Moreover, it has also been proposed to consider the heterogeneity of macrophage functions as a consequence of interaction with different immunological pathways (e.g., interaction with different growth and survival factors, interaction with lymphoid and myeloid cytokines, interaction with pathogens, resolution), rather than attributing them to distinct macrophages subsets (185).

In summary, the initial inflammatory response is carried out by activated macrophages in classical or alternative modality (depending on the triggering events), aiming at eliminating invading microbes by promoting the inflammatory response. Then, the resolution phase is carried out by macrophages in deactivated modality, unresponsive to inflammatory stimuli, and active in the elimination of the injured cells and tissue components, in promoting angiogenesis, cell proliferation, matrix deposition, and in general in tissue remodeling. The mechanisms that account for macrophage deactivation play a key role in maintaining homeostasis and keeping the immune response under control (189). Both innate and adaptive signals can influence the macrophage functional phenotype, which can have potentially dangerous consequences if not appropriately regulated. For example, classically activated M1 macrophages can cause damage to host tissues, predispose surrounding tissue to neoplastic transformation and influence glucose metabolism by promoting insulin resistance. Macrophages that are normally involved in wound healing can promote fibrosis, exacerbate allergic responses, and be exploited by pathogens for intracellular survival. These M2-type macrophages can contribute to the progression of neoplasia by promoting tumor survival (see Table 3).

### Current hypothesis on macrophage polarization

Plasticity and flexibility are key features of macrophages and of their activation states. A controversial issue is whether a phenotypic and functional evolution of macrophages occurs *in vivo*, and how it happens. As mentioned above, it has been observed in mice that the M1 to M2 switch during the progression of the inflammatory response enables macrophages to perform different activities in the different phases of the reaction. The controversy refers to the mechanisms underlying this switch, i.e., whether M1 and M2 macrophages are phenotypically distinct subpopulations that can serve different functions in different phases of an inflammatory reaction (45, 150), or the same cells can shift from one to another functional phenotype in response to microenvironmental signals (156, 157).

Several hypotheses are attempting to explain the issue. A first hypothesis is that different subsets of monocytes or macrophages can adopt a different functional phenotype. Thus, Ly6C^+^ monocytes and/or monocyte-derived macrophages in the tissue become M1 macrophages, and Ly6C^−^ monocytes and/or tissue-resident macrophages become M2 macrophages. It is possible that resident macrophages maintain cytoprotective and reparative functions, whereas macrophages derived from circulating inflammatory monocytes perform mainly M1 type functions. This hypothesis is not fully supported by the studies previously cited, where in different situations it was possible to observe both the differentiation of Ly6C^+^ cells in M1 and of Ly6C^−^ cells in M2 (45, 150) and the transdifferentiation from Ly6C^+^ M1 cells to Ly6C^−^ M2 cells (156, 157).

A second hypothesis is that there are sequential waves of monocyte recruitment into a tissue throughout the course of an inflammatory reaction. Therefore, monocytes recruited into the tissue at different times encounter different microenvironments with different signals that can polarize them in M1 during early phases and in M2 in late phases (156). In this case, cytokines and other microenvironmental signals in the tissue play a key role in determining the different functional phenotypes of macrophages. Although the role of cytokines in steering the macrophage functional phenotypes has been proven *in vitro* (179), the situation could be very different *in vivo*, where M2 activity is strongly increased in sterile wounds (157) or injured kidney (190) in absence of Th2-like cytokines IL-4 or IL-13 (which in any case do not induce the typical M2 phenotype, i.e., the deactivated healing/repairing functional phenotype). In these cases, M2 macrophages derive largely from M1 macrophages, with monocytes recruited from the circulation first acquiring an inflammatory phenotype, and then persisting in the tissue and maturing into repair macrophages.

Based on the latter data, a third hypothesis is that polarized macrophage populations can switch one to the other in response to different conditions. Data from *in vitro* studies demonstrate that human monocytes can acquire the phenotype of polarized M1 macrophages and then mature into M2 repair macrophages upon exposure in culture to sequential changes in the microenvironmental conditions (191). Other studies demonstrated that M2 macrophages are reprogramed to express M1 genes following exposure to TLR ligands or IFN-γ (192, 193).

A related question is whether both tissue-resident macrophages and monocyte-derived macrophages can polarize in M1 or/and M2 functional phenotypes. We have described above that tissue macrophages have basically an M2-like phenotype, whereas infiltrating recruited monocytes differentiate in M1 or M2 depending on the tissue conditions. For instance, it has been shown that tissue-resident macrophages, rather than recruited monocytes, are alternatively activated in the tissue during infection with *Litomosoides sigmodondis* (102). Also, recruited monocytes can be directly polarized into an anti-inflammatory M2 phenotype by basophil-derived IL-4, in order to alleviate allergic inflammation in the skin (194). Although it is not possible discriminating between tissue-resident and monocyte-derived macrophages in steady-state conditions, it seems that alternatively activated tissue macrophages have a transcriptional profile and phenotype different from that of alternatively activated monocyte-derived macrophages, with the latter having immunoregulatory properties (195).

It should be considered that *in vitro* studies do not fully recapitulate *in vivo* differentiation for two main reasons:

1. These studies are generally based on an heterogeneous population of monocytes, encompassing all the blood subsets (Ly6C^+^ and Ly6C^−^ in the mouse, and CD14^+^ and CD16^+^ in human being), thus it cannot be defined whether upon different stimuli the same cells can pass from a phenotype to another or whether different subsets are activated in response to different stimuli.
2. While M2 macrophages can convert to the M1 phenotype, the reverse generally does not occur, or it may only occur in particular conditions (e.g., in very mild inflammatory responses). In fact, M1 is probably an end-stage killer cell that dies during the inflammatory response, possibly succumbing to its own NO production, as it was demonstrated *in vitro* (196). So, their selective death may give the impression that they convert in M2 cells, which in fact proportionally increase (145). It seems that M1 vs. M2 polarization correlates with the capacity of macrophages to produce NO (166) as opposed to the important M2 driver TGF-β (164, 197, 198), thus the decrease in NO-producing macrophages would increase TGF-β production and amplify M2 polarization.

There are cases in which a phenotypic switch in the macrophage population occurs over time, often associated with pathology (91, 141). Three specific examples of this phenotypic switch are the following:

*endotoxin tolerance*, an altered state of responsiveness to secondary stimulation with LPS, resulting in a global and sustained switch of the gene expression program from an inflammatory M1 signature to an anti-inflammatory phenotype (199);
obesity-induced insulin resistance or type 2 diabetes, and atherosclerosis lesions. These are *metabolic syndromes* that can lead to a switch in the phenotype of adipose tissue macrophages from wound healing (as in healthy non-obese human beings) to classically activated macrophages (200, 201);
*cancer*, where the tumor-infiltrating classically activated macrophages have the potential to contribute to the earliest stages of neoplasia (202–204), and then, as the tumor progresses, can progressively differentiate to a regulatory phenotype and eventually become cells that share the characteristics of both regulatory and wound-healing macrophages (181).

Although the pathology provides the proof-of-principle that macrophages can undergo dynamic transitions between different functional states, it is possible that a mixture of M1/M2 phenotypes underlies these conditions (14, 145, 166). In the past few years, gene expression profiling techniques and genetic approaches have been used to cast some light on the plasticity of macrophage activation. The commonly held view is that macrophage polarization is driven by cues in the tissue microenvironment, which can include cytokines, growth factors, and microorganism-associated molecular patterns. These signals are thought to dictate a transcriptional response that shapes the phenotype and function of macrophages based on the physiological or pathological context. Progress has been made in defining the molecular mechanism underlying macrophage polarization, including signaling pathways, miRNA, epigenetic modification, post-transcriptional regulators, and transcriptional factors (189, 205–207). However, the data are still incomplete and far from being systematic, and our knowledge of the mechanistic basis of macrophage diversity in different tissues or in response to changing environment is to a large extent unknown.

## Post-Inflammation Fate of Monocytes/Macrophages

### Antigen presentation in non-lymphoid organs

The capacity of taking up and presenting antigen (i.e., the linking function between innate and adaptive immunity) is one of the most important features of tissue macrophages (208). It has been mentioned above that some monocytes that enter the tissue during inflammation do not differentiate into macrophages, and are able to take up antigen in the tissue and carry it to lymph nodes where they can present it to naïve T-cells (64). In addition to this population of monocyte-like cells, tissue macrophages are also able to present antigen, despite the fact that they do not recirculate to lymph nodes after antigen uptake. That tissue macrophages are highly phagocytic and can take up microorganisms and other matter in the tissue is well known, as this is their major function both in homeostasis and during inflammation. That antigen presentation may occur also in non-lymphoid organs has been suggested by several experimental evidence describing antigen-specific local activation and expansion of primed T-cells, but not of naïve T-cells (209–215). Based on this evidence, the hypothesis proposed by Ley is that initial priming of naïve T-cells occurs in the lymph node (to which antigen-loaded tissue monocytes recirculate), but that the full activation and effector functions of T-cells occur in the tissue where the inflammatory reaction is taking place, upon the productive interaction and formation of immunological synapse between primed T-cells and the antigen-presenting tissue macrophages (the difference between monocyte-derived tissue DC and tissue macrophages is bleared, as they seem to be not much more than slightly different functional differentiation states from a common precursor). Most likely, the inflammatory monocyte-derived cells with an M1-like functional phenotype are the antigen-presenting cells (APC) that induce activation/polarization of effector Th1 and Th17 cells upon production of IL-12 and IL-23, respectively, and in a TNFRSF and TNFSF-dependent fashion (but independent of CD80, CD86, and CD28 co-stimulation). Likewise, M2-like tissue macrophages, which produce TGF-β and express the αVβ8 integrin are likely involved in the polarization of iTreg cells, whereas their role in Th2 polarization is less clear (208).

### Fate of activated resident macrophages and recruited monocytes: Proliferation, replacement, and M2-like polarization

Based on what described above, the cell populations present in the tissue during the acute phase of an inflammatory reaction are the following:

- Tissue-resident macrophages and monocyte-derived macrophages. These, after initial recognition of microbial or damage-associated molecules, drive the influx of blood-derived monocytes, which will become inflammatory macrophages. Their role in initiating the inflammatory reaction possibly depends on the nature and grade of challenge.
- Monocyte-derived macrophages, newly recruited and rapidly occupying the inflammatory lesion, becoming the majority of the macrophages present in the tissue. These cells induce the inflammatory response by differentiating in the M1 functional phenotype.
- Tissue monocytes, the recently described cells that can take up antigens in the tissue and move to lymph nodes, where they are able to present antigens to naïve T-cells.
- Memory macrophages, or trained monocytes, cells functionally programed by a previously stimulus for either enhanced (training) or decreased (tolerance) cytokine production, depending on the type and concentration of the stimulus they encountered [(216); see below]. Here, we consider them as a kind of resident inflammatory monocyte-derived macrophage, able to react in a faster and stronger manner compared to other macrophages.

**Figure 4:**
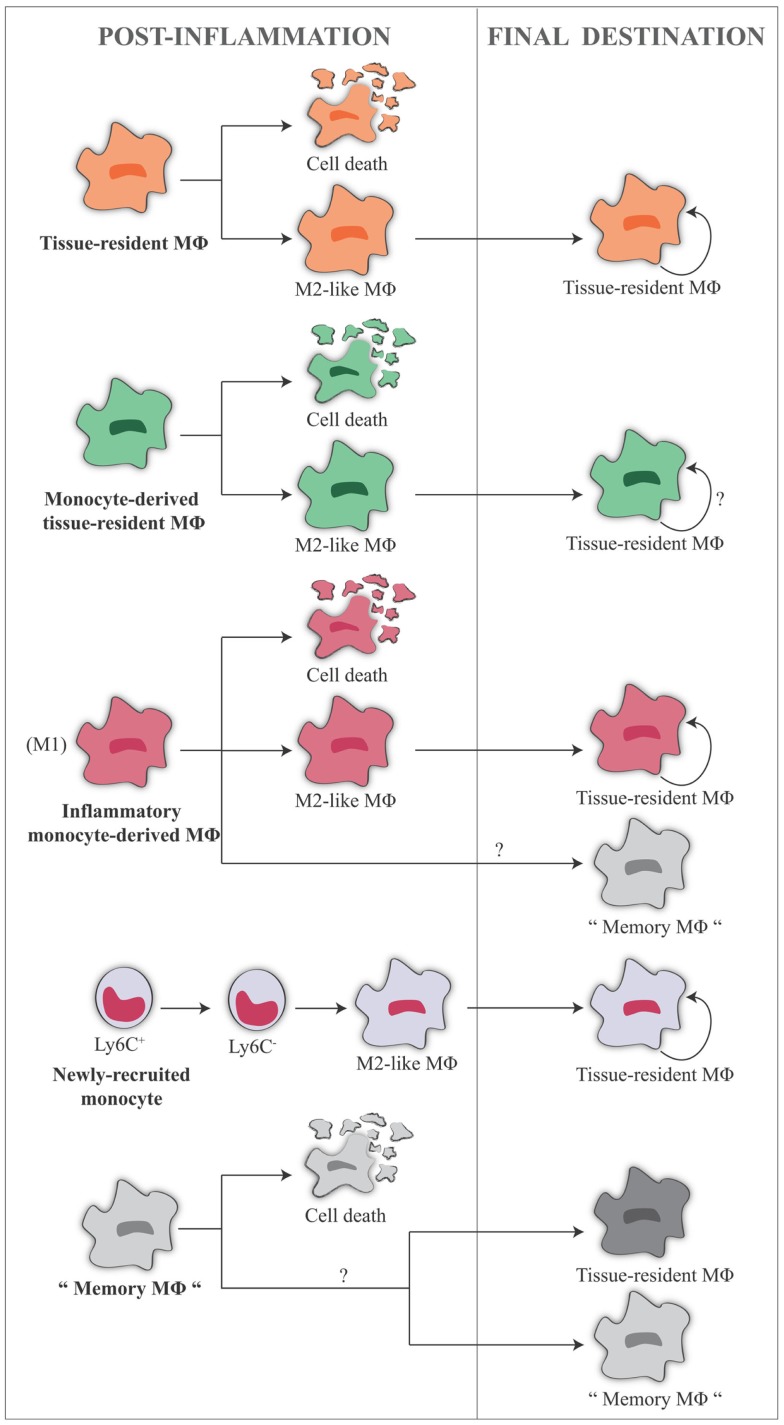
**Fate of the different monocyte/macrophage populations in the tissue during the post-inflammatory phase**. Tissue-resident macrophages are in general maintained locally by proliferative self-renewal, and retain an M2-like functional phenotype. The same situation is hypothesized for monocyte-derived resident macrophages, since it is not possible to fully discriminate between the two populations. A number of cells of these two populations probably die during the inflammatory reaction. Inflammatory monocyte-derived macrophages can die killed by the NO they have produced, and the surviving cells can undergo *in situ* phenotype conversion and become M2-like tissue-resident macrophages. In addition, a number of these cells can conserve a “memory” of their past inflammatory activation, and become trained monocytes/memory macrophages. Monocytes recruited from the blood during the post-inflammatory phase can lose the expression of Ly6C and become Ly6C^−^ cells, subsequently differentiating in M2 macrophages. They may also become memory macrophages. Memory macrophages that are present in the tissue, reminiscent of previous inflammatory events, would probably behave like naïve macrophages upon a new inflammatory challenge, except for a much quicker reaction, and will, therefore, mostly die or generate M2-like macrophages or again memory macrophages. Their life span in the tissue is presently unknown.

A summary of the different macrophage types and of their fate after the acute inflammatory phase is given in Figure 4.

In general, tissue-resident macrophages are maintained locally by proliferative self-renewal (100, 106), and retain an M2-like phenotype, for example, in the peritoneal cavity, brain, and lung (86, 100, 161). The fate of monocyte-derived resident macrophages is hard to follow, considering that it is not possible to fully discriminate between them. However, we may hypothesize that they have the same fate of tissue-resident macrophages, i.e., they maintain an M2-like phenotype and a low self-renewal capacity. A number of cells of both populations probably die during inflammation, the extent of their survival possibly depending on the nature and magnitude of the insult.

Generally, the inflammatory monocyte-derived macrophages are polarized toward M1, and the majority of them dies, killed by their own NO production (see above). In an experimental acute lung injury model, these cells undergo Fas-mediated death, while the resident alveolar cells persist (217). From that, we can argue that M1 likely is a terminal differentiation phenotype. However, there are reports that they can also undergo *in situ* phenotype conversation to become tissue-resident macrophages either during inflammation or after experimental deletion of tissue macrophages (48, 86). This underlines the notion that macrophage polarization is both transient and plastic.

The survival in the tissue of inflammatory monocyte-derived macrophages raises important questions that need to be answered.

Do monocyte-derived tissue macrophages conserve a “memory” of their past inflammatory activation, thereby becoming memory macrophages? And, do tissue macrophages resume their previous functional phenotype in response to a new inflammatory challenge? Or, do they react as naïve cells?

Memory macrophages (also recently termed “trained monocytes”) have been described, which retain a memory of past challenges (see below). Their fate in the tissue is, however, unknown, since no long-term experiments have been performed in mammals. It is possible that a part of them dies after reacting to a new inflammatory challenge. If some of them survive (again, this possibly depends on the type and magnitude of the new challenge), they would probably behave like inflammatory monocyte-derived macrophages, i.e., they could become M2-like cells, having a low level of self-renewal, and may also form a new population of memory macrophages that retain the memory of multiple challenges.

Another population that should be considered is that of monocytes recruited from the blood during the post-inflammatory phase. It is possible that these cells lose Ly6C expression when in the tissue, thereby becoming Ly6C^−^ cells that subsequently differentiate in M2 macrophages.

### Memory macrophages

It is long known that innate immune responses are higher to a secondary infection/challenge, and that this higher reactive occurs whether the new challenge is the same or different from the first one (cross-protection). An old example is that of mouse peritoneal macrophages from BCG-infected mice that have little/no activity 7 days after infection, and acquire significant citocydal activity upon *in vitro* challenge with LPS or with a wealth of other stimuli, while naïve macrophages do not (218). Recently, this phenomenon has been re-named *trained innate immunity* (219). Innate memory plays an important defensive role in organisms lacking adaptive immunity, such as plants and invertebrates, but it is evident also in vertebrates lacking functional T and B lymphocytes (220). In these animals, this innate memory mechanism was shown to involve innate immune cells with low turnover [such as macrophages and NK cells; (221, 222)] that would be responsible for improved pathogen recognition through pathogen recognition receptors, and for an enhanced protective inflammatory response (223, 224). NK cells could generate a memory response to viruses, while macrophages retain memory of both bacterial and viral challenges. A logical possibility is that the microorganisms encountered by the host on a regular basis may serve to differentiating and continually renewing a pool of memory-like macrophages with enhanced reactivity to infectious challenges. The molecular mechanisms responsible for shifting macrophages toward a memory status have not yet been elucidated. Putative mechanisms may involve differences in the monocyte/macrophage population (i.e., CD14^+^ and CD16^−^) or changes in the expression of lectin receptors on cell membrane (221), or in the functional phenotype (e.g., phagocytosis or protein production), but all are probably underlain by epigenetic reprograming that, through modification of DNA, post-translational modifications of histones (methylation), or microRNA, regulates gene expression by inducing dynamic alterations in the chromatin structure (220). Establishment of macrophage memory, depending on the experienced challenges, is likely to rely on epigenetic changes, as these can be at the basis of a rapid evolution of responsiveness and adaptation to incurring events, thereby allowing to surviving to new environmental threats (220, 225). Efficacy of many vaccines probably implies the induction of non-specific macrophage memory that contributes to the increased resistance to infections. Research in the field of memory macrophages needs a thorough re-assessment of a large body of old evidence accumulated in the past decades in the areas of macrophage activation and of adjuvanticity.

## Conclusion

An increasing amount of evidence supports four revolutionary concepts/discoveries on monocytes/macrophages that will force the researcher to rewrite the books of immunology:

1. The *embryonic origin of tissue-resident macrophages*, which raises the need to better understand the features/properties of monocytes (that are no longer simple precursors of tissue macrophages), and those of macrophages, which are capable of self-renewal without loss of their differentiated cellular identity.
2. The *capacity of monocytes/macrophages to polarize* into distinct functional phenotypes able to initiate and direct virtually all immune responses, including adaptive ones.
3. The notion of *innate memory*, an old concept that has been recently revived with the description of the so-called trained innate immunity.
4. The importance of *macrophage-mediated antigen presentation* in tissue responses, with the identification of antigen-uptaking, recirculating, and presenting “tissue monocytes,” and with the notion that tissue macrophages are probably the major APC upon a second challenge at the tissue level, without need of recirculation to the lymph nodes.

The central role of monocytes/macrophages in this new view of immunity implies that innate immunity has a major role in inducing and modulating adaptive immunity (including the induction of polarized T-cell responses), while on the other hand taking advantage of adaptive immune mechanisms (e.g., T-cell-derived cytokines) for modulating its own activity. Thus, new knowledge on macrophage biology and functions will have a direct impact on our understanding of immune responses and on the design of novel therapeutic strategies. For this reason, it is necessary to overcome several experimental obstacles that delay the full understanding of the new dynamics and relationships within the immune system, and that have been identified by the researchers cited in the review.

For example, to date, transcriptome analysis of monocyte subsets has been done at the basal unstimulated level, showing dramatic differences consistent with a different functional repertoire for the three types of human monocytes. Circulating monocytes are most likely “quiescent” (their quiescent status is needed in order to avoid developing a deleterious intravascular inflammation), while their effector functions only develop after relocation and activation in the tissue. Thus, the true role of the different monocyte subsets could be only understood after activation, and the stimulus-induced transcriptome of these cells will be required. Further, the models of inflammation used to test the proliferative capacity of resident macrophages have so far been limited to one or two rounds of tissue repopulation or relatively acute periods of infection/inflammation. This obviously cannot provide reliable information on the long-term capacity of macrophage self-renewal. Moreover, when studying the plasticity and interchangeability of M1 and M2 macrophages, since mixed M1/M2 phenotypes can be found especially in pathological conditions, it is capital to focus not only on populations but also either at the single cell level or by lineage-tracking studies (e.g., with mice expressing Cre ricombinase under the iNOS or arginase promoters, to track M1 and M2 lineages, respectively). Precautions need to be taken when drastic experimental procedures such as monocyte depletion or parabiosis are used to study macrophages self-renewal. These treatments can alter the concentration of circulating CSF-1 and CSFR1 signaling, which are important for self-renewal of resident macrophages under homeostatic conditions, and critical for differentiation of monocytes into tissue macrophages. Likewise, precautions and appropriate controls need to be implemented when using CCR2-deficient mouse for studying monocyte recruitment to the tissue, since the CCL2/CCR2 chemokine system is also responsible of the release of monocytes from bone marrow. Thus, the lack of recruitment of monocytes from the blood to the tissue could be due to lack of release of monocytes from bone marrow to the blood, where circulating monocytes are decreased.

Our final recommendation, therefore, is probably obvious, but it is anyway important to state it again. We need to re-evaluate patiently and critically a huge body of experimental evidence that is already present in the literature. In particular, we need to overcome the lack of consensus in defining and describing the different macrophage phenotypes (226). Many old studies have already generated information that, in light of our present knowledge, can become very important and help us to clarify the general picture. Second recommendation is that of designing experiments very carefully, keeping in mind that the immune system is redundant and that the same factor can have different activities, and that the same activity can be carried out by different factors. Third recommendation: monocytes and macrophages are never isolated in the body, and what they do and what they become are totally influenced by the surrounding cells and tissue. *In vitro* systems may only partially reproduce this complexity. Last recommendation: consider evolution as an incommensurable and most precious source of information that can greatly help us understand the ontology and behavior of monocytes and macrophages. Common mechanisms are many, and also species-specific differences exist, thus we should be able to pick up the relevant common information without, however, forgetting that human being is not a mouse or a mosquito.

## Author Contributions

Paola Italiani wrote the paper; Diana Boraschi contributed to writing and critically revised the paper.

## Conflict of Interest Statement

The authors declare that the research was conducted in the absence of any commercial or financial relationships that could be construed as a potential conflict of interest.
